# Shutoff of Host Gene Expression in Influenza A Virus and Herpesviruses: Similar Mechanisms and Common Themes

**DOI:** 10.3390/v8040102

**Published:** 2016-04-16

**Authors:** Hembly G. Rivas, Summer K. Schmaling, Marta M. Gaglia

**Affiliations:** 1 Department of Molecular Biology and Microbiology, Tufts University School of Medicine, Boston, MA 02111, USA; Hembly.Rivas@tufts.edu (H.G.R.); Summer.Schmaling@tufts.edu (S.K.S.); 2 Post-Baccalaureate Research Education Program, Tufts University School of Medicine, Boston, MA 02111, USA; 3 Graduate Program in Molecular Microbiology, Tufts University School of Medicine, Boston, MA 02111, USA

**Keywords:** host shutoff, herpesviruses, influenza A virus, Kaposi’s sarcoma-associated herpesvirus, herpes simplex virus, RNA degradation, transcription block

## Abstract

The ability to shut off host gene expression is a shared feature of many viral infections, and it is thought to promote viral replication by freeing host cell machinery and blocking immune responses. Despite the molecular differences between viruses, an emerging theme in the study of host shutoff is that divergent viruses use similar mechanisms to enact host shutoff. Moreover, even viruses that encode few proteins often have multiple mechanisms to affect host gene expression, and we are only starting to understand how these mechanisms are integrated. In this review we discuss the multiplicity of host shutoff mechanisms used by the orthomyxovirus influenza A virus and members of the alpha- and gamma-herpesvirus subfamilies. We highlight the surprising similarities in their mechanisms of host shutoff and discuss how the different mechanisms they use may play a coordinated role in gene regulation.

## 1. Introduction

During infection with many human viruses, the accumulation of viral proteins is accompanied by a progressive global reduction in the production of host proteins, a phenomenon that has been termed “host shutoff”. Viruses have evolved ways to interface with many, if not all, of the steps in gene expression to carry out host shutoff: transcription, messenger RNA (mRNA) processing, mRNA export from the nucleus, regulation of mRNA stability, and translation. As part of the evolutionary arms race between viruses and infected cells, host shutoff is thought to contribute to cellular takeover in two ways: By redirecting cellular resources towards viral gene expression and by promoting immune evasion. Moreover, host shutoff mechanisms may interfere with anti-viral stress responses.

In principle, host shutoff can free up machinery and thus redirect cellular resources towards viral gene expression. Reducing levels of cellular mRNAs or preventing their association with ribosomes and translation initiation factors can facilitate translation of viral mRNAs. Similarly, in the case of DNA viruses, suppressing transcription of host mRNAs may make general transcription factors and the RNA polymerase complex available for viral mRNA transcription. However, proving a causal relationship between host shutoff and increased access to cellular machinery has been challenging, and only a few studies have shown evidence of this happening [1,2].

In contrast, perhaps the main and best-demonstrated role for host shutoff is immune evasion and in particular subversion of early innate immune responses. Viruses are sensed by several cellular receptors that detect viral components, such as double-stranded RNA and DNA in the cytoplasm (reviewed in [3]). Once one of these sensors is activated and the virus is detected, the cell fights back by producing type I interferons (IFNs), IFN-α and IFN-β [4]. To counteract this host response, many viruses specifically interfere with the type I IFN signaling pathways and directly alter expression of IFNs or downstream IFN-stimulated genes [5]. However, global host shutoff also contributes to blocking IFN responses and affects the expression of a number of innate immune signaling proteins in many different viral infections. In addition, it can also reduce the expression of major histocompatibility (MHC) molecules and presentation of viral intracellular antigens [6,7], thus interfering with activation of the adaptive immune system.

Several recurring themes emerge when surveying the plethora of host shutoff mechanisms used by different viruses. First, many viruses have converged on similar molecular pathways to regulate host gene expression. This suggests that some steps in gene expression may constitute particularly vulnerable points in gene regulation that can be exploited by pathogens to subvert host function. Second, not only do many viruses carry out host shutoff, but also each virus often does so through multiple molecular mechanisms. This underscores the importance of manipulating host gene expression during viral infection. At present, we do not fully understand how multiple mechanisms used by the same virus are integrated, and whether they act redundantly as “fail-safes” or have specific roles at different stages of the viral replication cycle. Third, in a majority of cases, host shutoff mechanisms discriminate between host and viral gene expression, coordinately down-regulating the former while promoting the latter.

**Figure 1:**
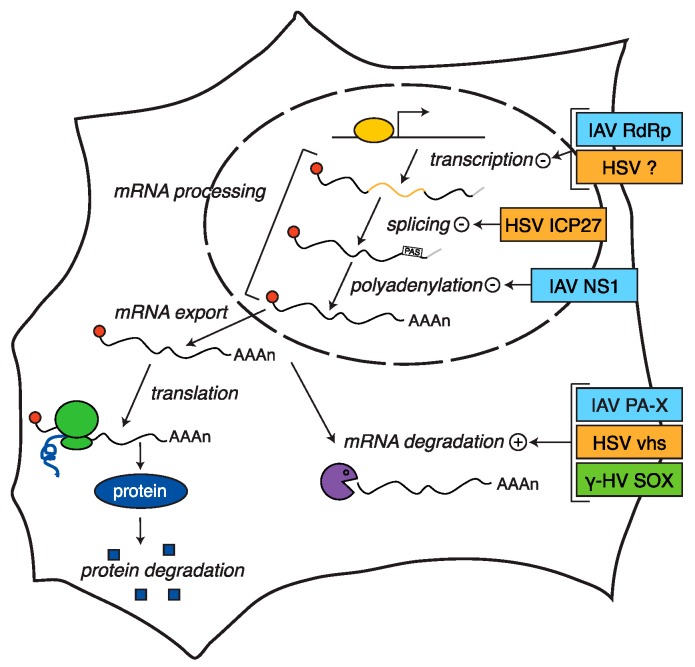
Herpesviruses and influenza A virus use multiple mechanisms to block host gene expression. In eukaryotes, production of proteins requires transcription, processing, nuclear export, and translation of mRNAs. The α-herpesvirus herpes simplex viruses (HSV), the γ-herpesviruses, and influenza A virus (IAV) use multiple viral factors to negatively regulate different stages of mRNA biogenesis and reduce host gene expression. They also stimulate host mRNA degradation.

In this review, we will discuss these themes in the context of the host shutoff mechanisms of the α-herpesviruses herpes simplex virus 1 and 2 (HSV-1 and -2), the γ-herpesviruses Kaposi’s sarcoma-associated herpesvirus (KSHV), Epstein Barr virus (EBV) and murine herpesvirus 68 (MHV68), and the orthomyxovirus influenza A virus (IAV). These viruses encompass many well-characterized examples of shutoff mechanisms (summarized in Figure 1) that are also used by other divergent viral families. We will discuss how, in spite of their considerable differences, these viruses use similar approaches to manipulate host gene expression at the transcriptional and post-transcriptional level, and employ multiple overlapping pathways for host shutoff.

## 2. Host Shutoff in Herpesviruses

Herpesviruses are large enveloped double stranded DNA viruses that encode more than 80 genes per virus. They are classified into three subfamilies (α-, β-, and γ-herpesviruses), based on differences in cell tropism. We will focus our discussion on α- and γ-herpesviruses because host shutoff has been extensively studied in these two subfamilies. α-Herpesviruses include the human viruses HSV-1 and -2, which cause oral and genital herpes. γ-Herpesviruses include the human viruses KSHV and EBV, which cause tumors of endothelial cells (KSHV) and B cells (KSHV, EBV), and the rodent virus MHV68, often used as a pathogenesis model. Two genetic programs define herpesviral infections: latent infection and lytic replication. During lytic replication all viral genes are expressed through a highly regulated temporal cascade characterized by immediate-early, early, and late viral genes. Herpesviruses depend on cellular transcription and translation factors to efficiently express their genes and replicate. Multiple mechanisms to directly regulate host gene expression have been described in α- and γ-herpesviruses, with global effects on transcription, RNA stability, and RNA processing. A role for these mechanisms in both immune evasion and viral protein production has been established.

### 2.1. Herpesvirus-Triggered RNA Degradation: Two Proteins, One Mechanism

The capacity to globally affect mRNA stability is a general feature of infection with several α- and γ-herpesviruses [7,8,9,10,11,12,13]. The best-studied host shutoff mechanism in HSV-1 and -2 and KSHV is mRNA degradation triggered by viral ribonucleases (RNases). The HSV protein UL41, or virion host shutoff (vhs), is an RNA endonuclease [14] of the FEN-1 nuclease family [15] that is packaged in the tegument (the protein layer) of infectious virions [16]. Homologs of this protein with RNA-degrading activity have also been described in other α-herpesviruses [12,13], but have not been studied as extensively. Release of vhs from virions during early infection induces accelerated degradation of many cellular [16,17,18], as well as viral, mRNAs [9,19,20]. This ability to degrade both host and viral transcripts enables vhs to not only control the necessary switch from host to viral gene expression, but also regulate viral mRNA levels in order to facilitate the shift between early and late viral gene expression [20]. Indeed, vhs mutations cause increased accumulation of early gene transcripts and a lag of late gene expression, instead of the typical cascade of viral gene expression necessary for efficient replication, thus leading to lower viral titers [8,19,20]. Notably, although vhs has only a small effect on virus production in cells, mutated vhs has a dramatic effect on virulence *in vivo*. HSV-2 carrying mutant or null vhs exhibit either attenuated or altered disease development in mice [21,22,23,24] due to increased host type I IFN responses [25,26,27]. These results led to the conclusion that vhs is essential for virus-host interaction, rather than viral replication *per se*, supporting a model of host shutoff as a means of immune evasion.

Interestingly, although lytic infection with γ-herpesviruses also triggers global degradation of host RNAs, these viruses use a different factor, encoded by the KSHV/MHV68 open reading frame 37 (ORF37), the γ-herpesvirus homolog of the herpesviral alkaline exonuclease (AE; the “alkaline exonuclease” name refers to the activity during DNA degradation *in vitro*, which requires an alkaline pH) [7,10,11]. The ORF37 protein is termed shutoff and exonuclease (KSHV SOX and MHV68 muSOX) and its EBV homolog is called BGLF5. Due to their PD-(D/E)XK nuclease fold and active site [28,29,30], the viral AEs have been classified as members of the type II restriction endonuclease-like superfamily [31]. In α-herpesviruses the AEs have an unclear role in the processing of the viral genome during viral DNA replication, but do not trigger widespread RNA degradation [10]. In contrast, the γ-herpesviral SOX family members have a dual function—a deoxyribonuclease (DNase)-mediated role in genome processing and an RNase activity involved in host shutoff [32]. Indeed, *in vitro* studies of EBV BGLF5 and KSHV SOX have shown that these proteins have both DNase and RNase activity [28,29], although they bind DNA substrates with greater affinity than RNA [29]. The same active site carries out both functions, as catalytically inactive mutants prevent both DNase activity *in vitro* and host shutoff upon SOX overexpression in cells [10,32,33]. However, the two functions are genetically separate, as demonstrated by the isolation of point mutations in KSHV SOX, MHV68 muSOX, and EBV BGLF5 that cause selective defects in either the DNase or shutoff activity [32,34,35,36]. Surprisingly, the single amino acid mutations that distinctly isolate one of the two functions span across the entirety of the protein, suggesting that the two functions do not require separate domains. The current model is that different binding surfaces and specific residues on the SOX proteins mediate differential association with unknown cellular co-factor proteins for genome processing and host shutoff. This idea is supported by data showing that the *in vitro* RNase activity of KSHV SOX is not affected by some of the mutations that block its host shutoff function in cells [29]. Additionally, the binding to DNA and RNA is likely mediated by specific residues that may only partially overlap. The two functions of SOX proteins are also spatially and temporally separated, as DNA processing must occur in the nucleus after DNA replication, while RNA degradation requires localization in the cytoplasm and starts earlier in the viral life cycle [11]. A MHV68 strain (MHV68 ΔHS) carrying the R443I mutation in muSOX, which blocks host shutoff but preserves the genome maturation function, has been used by the Glaunsinger lab to study the effects of γ-herpesvirus host shutoff *in vivo* [34]. Surprisingly, muSOX-mediated host shutoff is not required for the acute phase of infection in the mouse lungs, but is important for establishment of latency in the spleen [34], which occurs approximately two weeks after infection and is a typical characteristic of γ-herpesviral infection. A number of different processes may be at play, including a shift between the latent and the lytic cycle balance [34] and a cell-specific requirement for host shutoff in viral replication [37], which prevent trafficking of the virus to the lymph nodes and spleen from the initial site of infection. In addition, like vhs, muSOX and its homologs may have a role in regulating host immune responses as the virus traffics through the body, which is consistent with the regulation of multiple innate and adaptive immune pathways by EBV BGLF5 [35,38,39,40].

**Figure 2:**
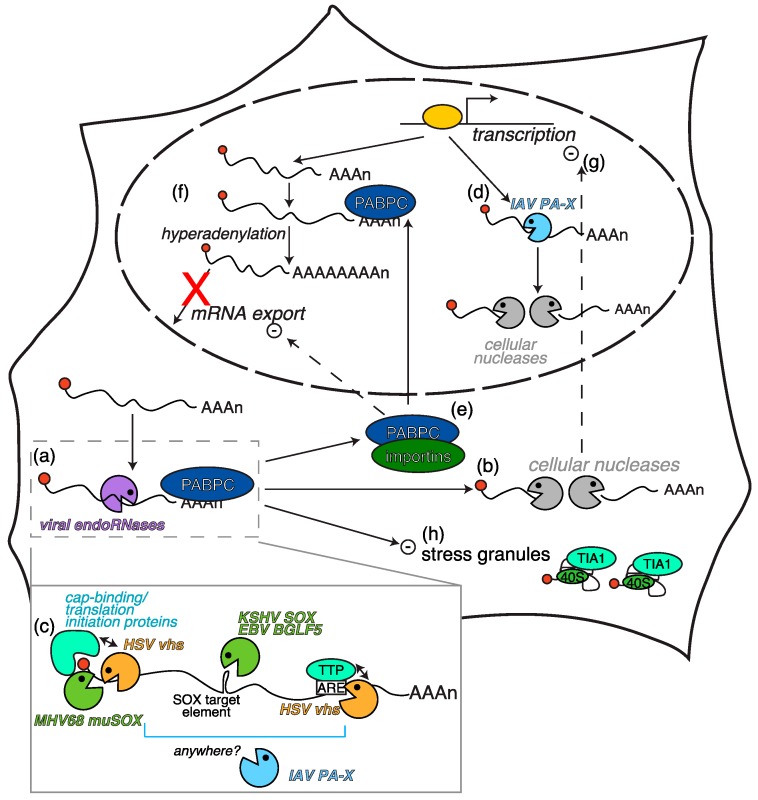
RNA degradation by herpesviral and IAV RNases and its downstream consequences. Viral host shutoff RNases cut mRNAs internally (**a**), leading to fragment degradation by cellular exonucleases (**b**). The location of the cut sites differs depending on the RNase ((**c**), inset). IAV PA-X also differs by potentially associating with targets in the nucleus (**d**). Secondary consequences of the RNA degradation include: (**e**) association of PABPC with importins and nuclear accumulation of PABPC, which results in mRNA hyperadenylation and export block (**f**); (**g**) a feedback inhibition of transcription mediated by the cellular exonucleases; (**h**) inhibition of formation of cytoplasmic stress granules.

Although α-herpesvirus vhs and γ-herpesvirus SOX are not homologs, they have a similar mechanism of action (Figure 2) [41], as they are both RNA endonucleases that cut host mRNAs into fragments. The initial cleavage is followed by degradation of the RNA body by the cellular 5′ to 3′ exonuclease Xrn1 and potentially other cellular enzymes [33,41]. This mechanism is also shared by non-herpesviral host shutoff RNases like IAV PA-X and SARS coronavirus nsp1 [2,41]. Moreover, all herpesviral host shut-off RNases, and in fact all currently known viral host shutoff RNases, degrade mRNAs transcribed by the cellular RNA polymerase II (Pol II) complex and spare non-coding RNAs (ncRNAs) transcribed by RNA Pol I (most ribosomal RNAs) and Pol III (small nuclear RNAs (snRNAs), transfer RNAs and other ncRNAs) [2,20,33,41]. However, vhs and SOX do not associate with Pol II-transcripts in the same manner and cut transcripts at different locations (Figure 2). This is not surprising, as the proteins have different sequences and structures.

Vhs cuts RNAs preferentially towards the 5′ end, in the translation initiation region of mRNAs [14,42], although some AU-rich elements containing mRNAs are cut in the 3′ untranslated region (UTR), the segment of RNA between the coding region and the 3′ end of the transcript [43,44,45] (Figure 2). Pol II transcripts are recognized by vhs because of its association with the cap-binding complex, which binds the 5′ 7-methyl-guanosine cap of mRNAs to initiate translation [46,47,48,49,50]. A yeast two-hybrid screen using vhs as bait initially demonstrated an interaction with the cellular translation factor eIF4H, a helicase accessory factor [46], and later studies also showed interactions with the entire cap-binding complex eIF4F [48] through the ATP-dependent RNA helicases eIF4AI and eIF4AII [47]. The interaction between eIF4H and vhs is essential for mRNA degradation because a vhs mutation (T214I) that abolishes direct interaction between vhs and eIF4H (but not eIF4F) abrogates vhs-mediated mRNA degradation in cells without affecting RNase activity *in vitro* [48,49]. Furthermore, knockdown of eIF4H prevents vhs-mediated RNA degradation [50]. Neither bacterially produced recombinant vhs, nor partially purified HSV virions extracts, recapitulate the selectivity of vhs for messenger *vs.* non-messenger RNAs seen in cells [46], indicating that interaction with cellular machinery is key for this protein (and likely every host shutoff RNase) to identify its physiological targets. A separate mechanism of RNA targeting has been described for mRNAs that contain AU-rich elements (AREs) in their 3′ UTR. AREs are bound by proteins that control recruitment of RNA degradation enzymes to the RNAs, regulating the stability of the transcripts [51], and are commonly found in mRNAs that code for immune-related genes like cytokines [52]. These ARE-containing mRNAs are cleaved by vhs within the 3′ UTR, and not in the 5′ region [43,44]. Direct binding of vhs to the ARE-binding protein tristetraprolin (TTP) may mediate this differential targeting of ARE-containing mRNAs [45]. Interestingly, a mutant of vhs that lacks a nuclear export signal and is trapped in the nucleus can still degrade ARE-containing RNAs, while it does not degrade other cellular mRNAs like GAPDH [53], underscoring the existence of different targeting mechanisms for these mRNAs.

In contrast to the position-directed specificity of vhs, KSHV SOX cuts RNAs in a sequence-specific manner [33,54] (Figure 2). This was first observed in reporter mRNAs, because knockdown of cellular exonuclease uncovered the presence of SOX degradation intermediates of defined and reproducible sizes [33]. This result was surprising because an RNA-seq study of cells transfected with KSHV SOX demonstrated that SOX expression alone reduces the levels, and presumably triggers degradation, of 60% of cellular mRNAs [55]. The widespread target range seems at first hard to reconcile with a sequence-specific cleavage mechanism. Nonetheless, the analysis of SOX cut sites using a transcriptome-wide approach revealed that sequence-specific cleavages are also present in host transcripts [54]. Furthermore, bioinformatics analysis identified a degenerate element that triggered SOX-mediated cuts when transplanted onto reporter RNAs [54]. This element included a few conserved sequence and structural features that could be widely found in host and viral mRNAs, explaining how SOX triggers widespread RNA degradation while eliciting sequence-specific cuts. It is currently unknown whether the SOX-targeting element is directly recognized by SOX or indirectly recruits the nuclease via a cellular factor. In the latter case, it is tempting to speculate that the SOX-interacting factor may be a ubiquitous cellular protein involved in mRNA biogenesis or metabolism, as this would offer a link to the selective targeting of Pol II transcripts. Reporter experiments have shown that EBV BGLF5 also cuts RNAs in a sequence specific way, but it is unclear whether this is true for MHV68 muSOX, because muSOX appears to cut reporter mRNAs at the 5′ end like vhs [41]. Additionally, the mechanism by which SOX and its homologs associate specifically with Pol II transcripts is not known, although SOX appears to be enriched in the monosome fraction in polysome profiling experiments [33], suggesting a direct link to translation.

How do viral transcripts escape RNA degradation by vhs and SOX? It would stand to reason that in the absence of a selectivity mechanism, host shutoff would not be beneficial for the virus. However, the mRNAs of DNA viruses are transcribed and processed by the same machinery as host mRNAs, and are generally very similar to host transcripts. Paradoxically, the answer may be that herpesviral mRNAs do not escape shutoff. In the case of α-herpesviruses, vhs actively degrades early viral transcripts, but it is then inactivated by other HSV proteins, VP16 [56,57,58,59] and VP22 [59,60], in order to allow production of late viral proteins. In fact, inactivation of vhs by VP16 and VP22 is required for viral replication [58,60]. Late viral mRNAs may also be directly resistant to RNA degradation [61] through unknown mechanisms. The viral protein UL47 may also have a role in protecting viral transcripts from degradation [62]. vhs degradation of early mRNAs is thought to facilitate the transition to late viral gene expression by getting rid of the early transcripts, and additionally by reducing “crowding” at the ribosome, shifting the balance from host to viral translation [1]. This is one of the better examples of a function for host shutoff proteins in the reallocation of gene expression machinery to viral processes. In γ-herpesviruses, there is no evidence that viral mRNAs escape shutoff, and, in fact, they are by enlarge upregulated during infection with the MHV68 ΔHS virus [37]. However, the virus appears to have evolved to adapt to host shutoff, because the composition of the virions is altered when host shutoff is inactive and the virus is less competent to replicate in murine embryonic fibroblasts and dendritic cells [37]. The altered balance of viral protein production in the ΔHS virus may underlie the cell-type specific replication defect and the trafficking defect of this mutant virus [34,37]. Similarly, EBV strains lacking BGLF5 make fewer virions, in part because the aberrant accumulation of some viral proteins prevents egress of viral capsids from the nucleus, where they are assembled [63,64]. This demonstrates that EBV BGLF5 normally also affects the expression of viral proteins, although in principle this could also be due to the DNase activity of BGLF5, because the mutants used in these studies were not single-function mutations.

KSHV causes three types of human tumors in immuno-compromised individuals, the endothelial cell-based Kaposi’s sarcoma and two rare B-cell lymphomas, primary effusion lymphoma and Multicentric Castleman’s disease. During development of KSHV-induced tumors, lytically infected cells are thought to be important because they produce inflammatory and angiogenic mediators that promote tumor formation and maintenance [65,66]. How do these cellular products still get produced during active host shutoff? Several studies have shown that some host mRNAs escape host shutoff [55,67]. A well-characterized “escapee” is the mRNA for the cytokine interleukin 6 (IL-6), which is important for viral growth and pathogenesis because IL-6 promotes the survival of KSHV-infected B cells [68,69]. The IL-6 mRNA is not degraded by SOX [67] because of its association with several cellular factors including the ARE-binding proteins HuR and AUF1 [70] and the multifunctional protein nucleolin [71]. Protective complexes assemble on a 200-nucleotide element in the IL6 3′ UTR, which can confer protection when fused to reporter genes [70,71]. Interestingly, the SOX-resistant element and nucleolin binding also protect RNAs against the action of other viral RNases like vhs [71]. Whether this protective mechanism extends to other host or viral mRNAs is currently unknown. For example, the cellular mRNA apoptosis enhancing nuclease (AEN) is also resistant to SOX-mediated degradation, but the mechanism of resistance does not rely on a 3′ UTR element and is currently unknown [55]. Moreover, the existence of a SOX-targeting element [54] suggests that some mRNAs may be naturally resistant to SOX cleavage. Whether this plays any role in gene regulation by the KSHV RNase remains to be seen.

Notably, although the SOX homologs in HSV-1 and -2, termed AE or UL12, do not cause global host shutoff by triggering mRNA degradation, as mentioned above, they specifically prevent expression of host genes from mitochondrial DNA (mtDNA) [72,73]. This effect is mediated by an *N*-terminal truncated version of the AE, UL12.5, encoded by the UL12 gene through the use of an alternative promoter, which results in expression of shorter 3′ coterminal mRNA [74]. Unlike full-length UL12, which is nuclear [75], UL12.5 is localized to the mitochondria and triggers mtDNA degradation [72,76]. Surprisingly, this is not directly due to its nuclease activity but is mediated by activation of the mtDNases ENDOG and EXOG [77]. UL12.5 is not required for replication of the virus in tissue culture [73,78], but affects type I IFN responses that are triggered by mitochondrial stress during HSV-1 infection [79]. Surprisingly, the presence of UL12.5 may increase rather than decrease the host innate immune response [79]. The mtDNA-depleting function of the AE is unique to HSV-1 and -2, because there is no evidence of expression of UL12.5-like truncated proteins or mtDNA degradation when homologs of UL12 from other herpesviruses are expressed [73]. It is interesting to note how the AE proteins of herpesviruses have acquired multiple additional functions in virus-cell interaction that are distinct depending on the virus.

### 2.2. Secondary Consequences of RNA Degradation by Vhs and SOX

Interestingly, widespread RNA degradation by α-herpesvirus vhs and γ-herpesvirus SOX and its homologs has several secondary consequences that potentiate the host shutoff effects (Figure 2). RNA degradation triggers nuclear relocalization of the cytoplasmic poly(A) binding protein PABPC [36,80,81]. PABPC is a nucleocytoplasmic shuttling protein that predominantly localizes to the cytoplasm, where it binds the poly(A) tails of mRNAs and mediates their translation [82]. However, during cellular stress like heat shock, oxidative stress, or transcriptional block, PABPC preferentially accumulates in the nucleus [83,84]. Recovery from heat shock returns PABPC to its preferred cytoplasmic localization [83]. In contrast, the PABPC nuclear accumulation caused by viral infection never resolves and persists until cell death [34,80,84]. Kumar *et al.* [85] discovered that KSHV SOX-mediated RNA degradation triggers relocalization of two isoforms of PABPC, PABPC1 and PABPC4, by unmasking a non-canonical nuclear localization signal (NLS). Like the canonical NLS, this region mediates interactions with the cellular adaptors importins α, which facilitate transport of proteins into the nucleus through the nuclear pore (reviewed in [86]). Although PABPC possesses no predicted canonical NLS, it can interact with several importin α isoforms via its four RNA recognition motifs (RRMs), as shown by *in vitro* pull-downs with recombinant PABPC and importins [85]. Moreover, deletion of all four RRMs prevents nuclear relocalization in the presence of SOX and *in vitro* association with importins [85]. The interaction between PABPC and importins is directly regulated by PABPC binding to the poly(A) tails of cytoplasmic mRNAs. Addition of poly(A), but not poly(C), RNA completely blocks PABPC interaction with importin α3 *in vitro*, while treatment with RNase I_f_ prior to pull-down enhances PABPC-importin binding [85]. Thus, during widespread viral-mediated mRNA degradation, the decay of cytoplasmic transcripts releases poly(A)-bound PABPC, exposing the RRMs, which then interact with importins. This process mediates PABPC relocalization to the nucleus. As vhs and the IAV RNase PA-X cause a similar localization of PABPC [2,81,87], it is very likely that an analogous mechanism is at play during HSV and IAV infection. Accumulation of PABPC in the nucleus directly promotes cellular poly(A) polymerase-dependent hyperadenylation of host transcripts as well as a nuclear mRNA export block [80,81], although it is unclear whether the hyperadenylation is the cause or the effect of the export block. Nonetheless, ultimately, a reduction in mRNA export likely contributes towards repression of host protein production by preventing the cytoplasmic pool of mRNAs from being replenished after degradation.

Feedback inhibition of host gene transcription has also been reported as an additional secondary consequence of RNase-triggered host shutoff. Infection with KSHV or MHV68 and overexpression of muSOX or vhs alone cause a reduction in transcription rates, measured by RNA metabolic labeling with 4-thiouridine (4sU), and a loss of RNA Pol II binding to host promoters [88]. Interestingly the transcriptional inhibition is dependent on the cellular RNA degradation machinery, which completes RNA degradation in the presence of viral RNases, because knockdown of the cellular RNA exonucleases Xrn1 and Dis3L2 blocks the feedback loop [88]. Because the cell senses the exonucleolytic degradation of the RNAs, rather than the virus-induced RNA fragmentation, this feedback is likely a homeostatic cellular response that detects altered RNA degradation as a sign of infection or stress. In contrast to the primary effect of RNA degradation, viral transcripts do escape this phase of host shutoff, and are transcribed as well or better during host shutoff in both MHV68- and KSHV-infected cells [88]. How the transcription of viral genes is protected is not currently understood. Nonetheless, the selective transcription of viral mRNAs during host shutoff by SOX and muSOX may provide a mechanism for γ-herpesviruses to compensate for the decreased stability of its own mRNAs.

The effects on PABPC localization and transcription are the main effects of SOX on stress responses. In the case of vhs, recent studies have shown that this protein also directly inhibits cellular stress responses by preventing the formation of stress granules (SGs) [89,90]. SGs are messenger ribonucleoprotein complexes (mRNPs) composed of mRNAs associated with stalled translation factors [91]. They are thought to provide a mechanism for the cell to store a pool of ready-to-go translationally inactive mRNAs during periods of stress [91] and to act as an anti-viral response to block translation of viral mRNAs during infection [92]. Although stress granules are not visible in cells infected with wild-type HSV-1 and -2, infection of vhs-deficient HSV-1 or -2 leads to SG formation [89,90], which is accompanied by reduced levels of late viral proteins, leading to attenuated virion production [89]. HSV-2 infection also inhibits SGs induced by exposure to sodium arsenite [93]. The SGs formed in virus-infected cells contain herpesviral proteins [90], although the significance of this finding is unknown. How vhs aids in the disruption of SGs is currently unknown, although the reduction in the cytoplasmic mRNA pool may be part of the effect. It is also possible that SG formation is a consequence of overloading of the translational machinery during late viral protein production, because vhs is thought to facilitate translation of late viral proteins by reducing the translational load on the ribosome [1]. However, the fact that knockout of T cell internal antigen 1 (TIA-1), a component of SGs, enhances HSV-1 replication [94] suggests that SG inhibition may constitute direct inhibition of cellular stress responses. Given the link of vhs activity to translation, it would be interesting to know whether vhs-mediated RNA degradation prevents SG formation or, alternatively, leads to rapid dissolution of the complexes due to loss of their mRNA substrates.

### 2.3. Other Host Shutoff Mechanisms in γ-Herpesviruses?

SOX and its family members are the only proteins identified as global host shutoff mediators in γ-herpesviruses at present. However, expression of SOX in uninfected cells [55] does not recapitulate the effects on host mRNA levels that are seen in KSHV-infected cells, in terms of both specific targets and magnitude [95]. This suggests the existence of additional, but unknown, host shutoff mechanisms in KSHV. The viral transcription factor K8α interacts with regulators of chromatin remodeling like the histone demethylase JMJD2A to regulate viral gene expression and has also been reported to globally reduce host mRNA levels upon overexpression in uninfected cells, presumably by altering transcription [96]. However, whether this occurs in infected cells is currently unknown. As discussed below, many viruses including HSV and the theoretically less complex IAV (see Section 3), use multiple mechanisms to control host gene expression, including increasing RNA degradation, altering RNA processing, and disrupting transcription. Thus, it is likely that γ-herpesviruses also harbor additional global host shutoff proteins, perhaps with more subtle effects than SOX.

### 2.4. HSV ICP27 Inhibition of RNA Splicing

**Figure 3:**
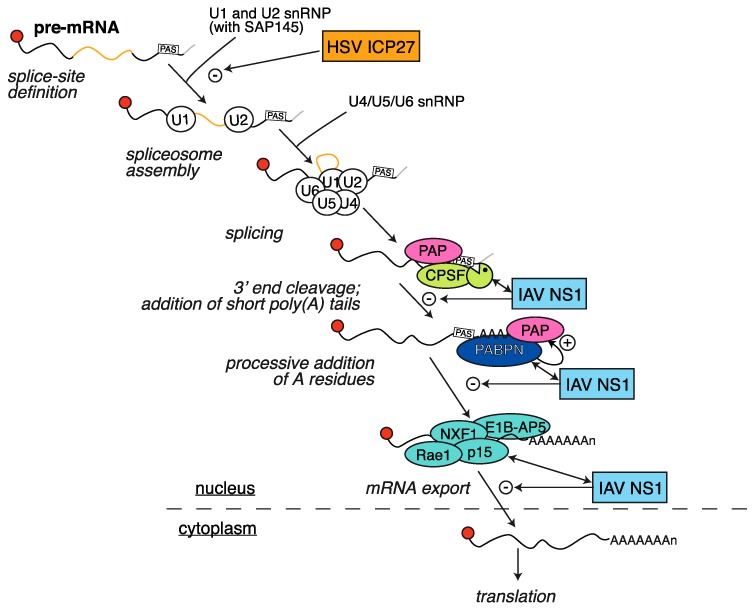
HSV ICP27 and IAV NS1 block mRNA processing. Newly transcribed pre-mRNAs are processed by splicing, that is the removal of introns (in yellow) through the action of the spliceosome complex. HSV ICP27 blocks the first step of complex assembly at splice sites. The pre-mRNAs are also processed at their 3′ end by the cleavage and polyadenylation complex (CPSF), which cleaves the RNA downstream of the polyadenylation signal (PAS) and allows poly(A) polymerase (PAP) to add short poly(A) tails. Nuclear PABPN is then required for stimulation of processive PAP activity and addition of the full-length poly(A) tails. IAV NS1 interferes with both steps of the process. For simplicity, splicing and 3′ end processing are represented in sequence, but they may occur simultaneously and also in part co-transcriptionally. IAV NS1 also interacts with components of the nuclear mRNA export complex (NXF1, p15, Rae1 and E1B-AP5) and blocks mRNA export.

In contrast to γ-herpesviruses, the existence of additional host shutoff mechanisms is well documented in HSV. The multifunctional immediate-early protein ICP27 has roles in transcription and post-transcriptional modifications of cellular transcripts, as well as promoting many aspects of viral gene expression (reviewed extensively in [97,98]). ICP27 inhibits host gene expression by decreasing levels of cellular mRNAs specifically in the cytoplasm [99]. This effect is due to the fact that ICP27 interferes with splicing of unprocessed intron-containing pre-mRNAs into mature mRNAs [100], and spliced mRNAs are exported more efficiently than unspliced mRNAs [101] (Figure 3). Although most of the studies have focused on HSV-1 ICP27, recent data shows a similar effect of HSV-2 ICP27 on splicing of host and viral mRNAs [102,103]. Formation of the spliceosome complex is strongly inhibited in the presence of ICP27 or HSV-1 splicing extracts [104,105], and ICP27 interacts with and regulates multiple splicing factors, including SRp20 [106] and SAP145 [105]. SAP145 is responsible for tethering the spliceosome complex A, which recognizes the splice sites and is composed of the U1 and U2 small nuclear ribonucleoprotein (snRNP) complexes [107]. SRp20 is a member of an essential group of serine-arginine-rich splicing factors termed SR proteins [108] that are involved in constitutive and alternative splicing, and are required for spliceosome assembly [109]. Interactions with SAP145 cause inhibition of spliceosome formation and splicing before the first catalytic step [105], interfering with formation of complex A. In addition, ICP27 induces hypophosphorylation and inhibition of SR proteins including SRp20 [106]. Normally, SR protein phosphorylation regulates splicing activity [110]. ICP27 interacts with SR protein kinase 1 (SRPK1), driving its relocalization to the nucleus and directly interfering with its kinase activity [106]. Thus, ICP27 suppresses splicing by preventing phosphorylation of SR proteins and abolishing formation of the cellular spliceosome.

The inhibition of splicing due to ICP27 is naturally selective for host transcripts, as most HSV transcripts are intronless. However, recent results using 4sU labeling of nascent RNAs (reviewed more extensively in the next section, [111]) have called into question how pervasive the effects of HSV-1 infection are on host mRNA splicing. Rutkowski *et al.* [111] did not detect widespread changes in splicing in cells infected with HSV-1. Nonetheless, the study did uncover that specific genes were spliced differently during HSV infection, suggesting that ICP27 has a key role in controlling alternative splicing.

### 2.5. Transcriptional Shutoff and Termination Defects during HSV-1 Infection

**Figure 4:**
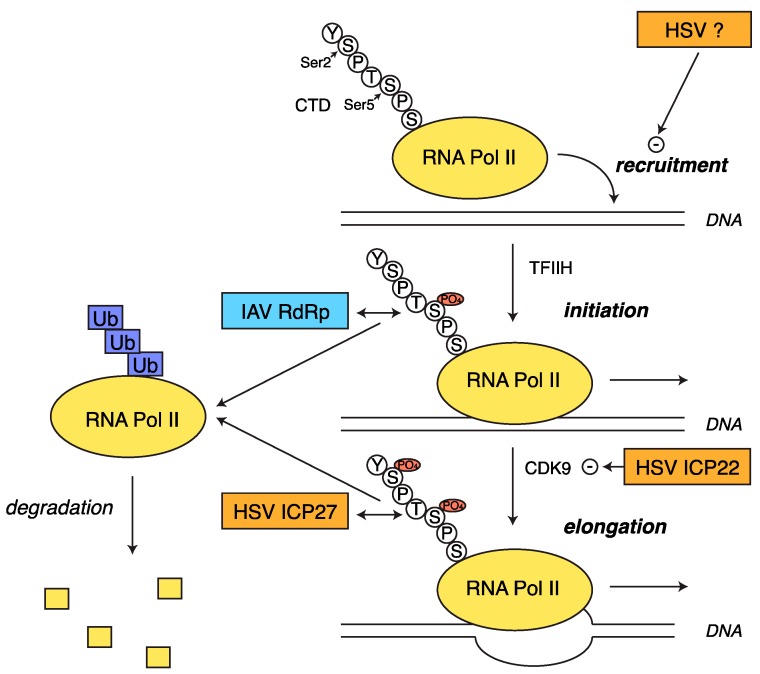
Both HSV-1 and IAV block host transcription. Transcription by RNA polymerase II (Pol II) consists of multiple steps: recruitment to the DNA, initiation, mediated by phosphorylation of the serine 5 (Ser5) of the *C*-terminal domain (CTD) of the large subunit of the Pol II complex by TFIIH, and elongation, mediated by phosphorylation of Ser2 of the CTD by cyclin-dependent kinase 9 (CDK9). Both ICP27 and the IAV RNA-dependent RNA polymerase cause ubiquitination (Ub) and proteasome-mediated degradation of Pol II, but may associate with different phosphorylated forms of the complex. HSV also blocks Pol II recruitment to the DNA via unknown factors and CDK9 activity via ICP22.

Although the RNA-degradation factor vhs and the splicing-inhibiting factor ICP27 are generally considered the main host shutoff proteins in HSV, it has been clear from early studies that transcription of host genes is also strongly repressed during HSV-1 infection [112,113]. Recently, high throughput sequencing-based studies have reiterated these findings, and have demonstrated pervasive changes in the biogenesis of host RNAs during HSV-1 infection by characterizing the DNA binding of host RNA Pol II and transcriptional rates at various times following infection [111,114]. Chromatin immunoprecipitation followed by deep sequencing (ChIP-seq) data revealed a severe reduction (98%) of Pol II binding, both at the promoter and along the whole gene body, for thousands of host genes, as early as four hours post infection [114]. This result indicates loss of actively transcribing Pol II as a consequence of reduced recruitment of Pol II to promoters, rather than reduced efficiency of transcription elongation (Figure 4). Interestingly, loss of Pol II occupancy was not observed on viral genes, which are also transcribed by RNA Pol II; rather Pol II occupancy was robustly increased across the HSV-1 genome [114]. The difference in Pol II occupancy between viral and host genes suggests that HSV-1 directly and selectively regulates Pol II activity, although it is not clear at present how the virus achieves this selectivity. Similarly, 4sU labeling of nascent RNA revealed that the transcription of 75% of cellular protein-coding genes is reduced two-fold or more during HSV-1 infection [111], demonstrating that Pol II is in a repressed transcriptional state. Surprisingly, the 4sU-labeling also uncovered the presence of intergenic transcription past the annotated 3′ end of thousands of host genes, indicating an additional defect in transcription termination that is specific for host mRNAs [111]. These longer transcripts are transcribed but do not associate with ribosomes [111]. Neither study identified the mechanism controlling transcriptional repression and suppression of transcription termination, although the transcription termination defect is not dependent on the known host gene regulators vhs or ICP27 [111]. It is possible that the transcriptional shutoff is analogous to the feedback transcriptional inhibition reported during muSOX-mediated host shutoff in MHV68-infected cells [88], although the timing may suggest otherwise.

Studies on Pol II phosphorylation during HSV-1 infection may offer an explanation for the suppression of transcription termination and Pol II occupancy. The *C*-terminal domain (CTD) of the large subunit of the RNA Pol II complex includes a series of Tyr-Ser-Pro-Thr-Ser-Pro-Ser tandem repeats that are differentially phosphorylated on the serines (Serine 2, 5, and 7) depending on whether the polymerase is clearing the promoter, elongating, or terminating [115]. HSV-1 infection alters the phosphorylation status of the repeats in the CTD of RNA Pol II. In particular, loss of phosphorylation at the serine 2 (Ser2) of the CTD may contribute to shutoff of host transcription during HSV-1 infection. Ser2 phosphorylation during the post-initiation stage of transcription enables Pol II to become elongation-competent and recruit splicing and polyadenylation factors [115]. In contrast to mock infected cells, where the elongating form of Pol II bearing phosphorylation on both Ser5 and Ser2 is predominant, in HSV-1-infected cells a Ser5-only form is predominant [116,117]. This shift in Pol II phosphorylation is due to the action of immediate early genes, because it occurs upon infection with a ICP4 mutant virus, which is unable to express delayed early or late genes [117]. Subsequent studies implicated the viral proteins ICP22 [118] and ICP27 [119] in this process (Figure 4). ICP22 is responsible for loss of Ser2 early during infection through its physical association and inhibition of cyclin-dependent kinase 9 (CDK9), which phosphorylates the CTD at Ser2 to promote elongation [120,121]. Because the CDK9 inhibition maps to a small domain in ICP22 that is conserved among *α*-herpesviruses [121], it is likely that other α-herpesviral homologs of this protein have similar effects. In addition, ICP22 was required for the enhanced appearance of the Ser5-only Pol II isoform [117], suggesting that Ser5 phosphorylation during infection may not be equivalent to Ser5 phosphorylation in resting cells. This is important in light of the Pol II occupancy results, as loss of Ser2 phosphorylation alone would be predicted to lead to increased paused polymerases, whereas loss of Pol II recruitment to transcriptional units was observed. In contrast, ICP27 promotes proteasome-mediated degradation of Pol II later in infection [119], removing both the hypophosphorylated and the Ser2 phosphorylated form. It is tempting to speculate that the loss of Ser2 phosphorylation may underlie the transcription termination defect, because some studies have shown that Ser5-phosphorylated Pol II can be transcriptionally active on human genes, but that the transcribed RNAs are not properly processed [122,123].

### 2.6. Herpesviruses: All Hands on Deck to Regulate Host Gene Expression?

In summary (Table 1 and Table 2), herpesviruses encode multiple viral proteins that control gene expression. Given the complicated replication cycle of herpesviruses and the highly regulated cascade of lytic gene expression, it is likely that the multiple mechanisms may provide different functionalities at different steps of the cycle. Transcriptional shutoff and RNA degradation may collaborate to definitively shut off induction of immune response genes early during infection, and RNA degradation may additionally clear existing RNAs in the cell, preventing new protein production. Processing changes may subtly modify the host transcriptome, and in the case of transcription termination defects, perhaps be an unintended consequence of remodeling the host machinery by shifting the balance of Pol II phosphorylation to suit viral transcription. Our understanding will be greatly improved by systematically combining high-throughput techniques with viral genetics, something that several labs have already started to do in the case of HSV, in order to dissect the contribution of different proteins and processes to host gene regulation.

## 3. Host Shutoff in Influenza A Virus

Influenza A virus (IAV) is a negative strand RNA virus of the Orthomyxoviridae family that replicates in the nucleus of host cells. Its genome encodes 14 proteins, several of which are synthesized through non-canonical translational and processing events [124]. Because IAV is a rapidly mutating virus, many strains of IAV have been isolated over the years. Studying the biology of this virus is complicated by the fact that many non-essential functions that modulate virus-host interaction vary between these strains. Nonetheless, global inhibition of host protein expression has consistently been observed in IAV-infected cells, starting with early studies of protein production using metabolic labeling (for example, [125]). Since then, multiple mechanisms of host shutoff have been described in IAV-infected cells, which is surprising given the small number of proteins encoded by this virus. Although early studies suggested a translational block may account for the loss of host protein production during IAV infection, more recent studies have focused on three main mechanisms of host shutoff by IAV infection: a block in cellular mRNA processing and nuclear export, degradation of host RNA Pol II, and widespread host mRNA degradation.

### 3.1. Inhibition of Polyadenylation and Export of Host mRNAs by NS1

Nonstructural viral protein 1 (NS1) is a multifunctional protein involved in several processes that inhibit the type I IFN responses (reviewed extensively in [126]). It is comprised of an RNA binding *N*-terminal domain and a *C*-terminal effector domain [127]. The crucial role of NS1 in counteracting the host innate response to IAV is apparent from studies on the infectivity of viruses that do not express NS1 (delNS1) [128,129]. delNS1 viruses induce high levels of IFN-α-stimulated reporter genes and replicate more efficiently in IFN-α/β-deficient cells (Vero cells) than in cells that express type I IFNs like MDCK cells [128,129]. Moreover, the delNS1 virus is not lethal in mice unless the animals carry a null mutation in STAT1, a key component of the IFN signaling cascade [129]. One of the mechanisms contributing to type I IFN inhibition by NS1 is a block in the nuclear processing of RNA Pol II transcribed RNAs [130,131,132,133] (Figure 3). Nemeroff *et al.* [132] showed that NS1 blocks the processing of 3′ end of cellular pre-mRNAs by inhibiting the cellular machinery that carries out this process. Pull down assays showed that NS1 exists in a complex with CPSF30, an essential component of the 3′ end processing machinery, the cellular cleavage and polyadenylation specificity factor (CPSF) complex. The CPSF complex is responsible for cleaving pre-mRNAs downstream of the polyadenylation signal (PAS) during transcription and recruiting poly(A) polymerase (PAP) to add the poly(A) tail to the 3′ end of the mRNAs (reviewed in [134]). NS1 inhibits CPSF activity, thus blocking polyadenylation of nascent cellular transcripts (Figure 3). Because polyadenylation and RNA processing in general are coupled to export of the mature mRNAs from the nucleus, NS1-mediated processing defects inhibit the nuclear export of these messages [130,131,132] and thus their access to translating ribosomes. To further ensure that any mRNAs that have escaped NS1 disruption of polyadenylation do not mature properly, the effector domain of NS1 also binds nuclear poly(A) binding protein (PABPN or PABII) and prevents it from stimulating the processive synthesis of long poly(A) tails catalyzed by PAP [133] (Figure 3). This leads to the accumulation of species with short (~10 nt) poly(A) tails that are exported less efficiently [133]. In addition to interfering with nuclear export of mRNAs indirectly by preventing correct 3′ end processing, NS1 also directly blocks mRNA export (reviewed in [135]) by interacting with cellular proteins involved in this process: NXF1, p15, Rae1 and E1B-AP5 [136]. These proteins form a complex that interacts with both mRNA and nucleoporins to direct mRNAs through the nuclear pore complex [137]. The direct inhibition of mRNA export is reversed by overexpressing NXF1, suggesting that NS1 is altering the nuclear mRNA export complex or preventing its association with mRNAs [136]. Moreover, NS1 also weakly interacts with and promotes degradation of the nucleoporin Nup98, which likely contributes to export defects [136]. Interestingly, chemical agents that affect the NS1-mediated nuclear export block have been identified. In particular, compounds that inhibit the *de novo* pyrimidine biosynthesis pathway restore mRNA nuclear export during infection or NS1 overexpression, and also inhibit IAV replication [138]. These compounds increase the protein levels of NXF1, suggesting that they might act similarly to NXF1 overexpression [138]. At present, it is not known how the direct nuclear export block and the polyadenylation block mediated by NS1 are connected.

In contrast to the mRNAs of DNA viruses like herpesviruses, the biogenesis of IAV mRNAs is very different from that of host mRNAs and requires virus-encoded machinery. Unlike host mRNAs, viral mRNAs are not processed by the CPSF complex. Instead, their poly(A) tail is added by stuttering of the viral RNA-dependent RNA polymerase (RdRP) on a polyuridine stretch on the viral genome segments [139]. Similarly, at least some of the IAV mRNAs may not require the NXF1-dependent pathway to be exported (reviewed in [135,140]). This makes viral mRNAs naturally resistant to NS1-mediated shutoff.

Interestingly, NS1-mediated host shutoff is not ubiquitously used by IAV strains. Particularly, strains of avian and swine origin, the mouse-adapted laboratory strain A/PuertoRico/8/1934 (PR8) and the human pandemic 2009 H1N1 strains, which have an NS1 of swine origin, carry NS1 proteins that do not inhibit 3′ end processing [141,142,143,144,145]. The structure of the NS1 effector domain in complex with CPSF30 indicated that amino acids 103 and 106 are key for NS1-CPSF30 interaction [146], and the identity of these residues vary between CPSF30-blocking and non-blocking strains [141,142,145]. Several studies have shown that reverting those amino acid to the consensus sequence (F103 and M106) in strains of avian and swine origin increases the pathogenicity of the virus in mice, suggesting that they could be important mutations in the process of human adaptation of different IAV strains [145,147]. To date, no study has tested the full extent of the effect of NS1 on host gene expression, and it will be interesting to see whether the effects of NS1 on IFN mRNAs extend to all host genes, or whether specific RNAs are more sensitive to NS1.

### 3.2. RNA Pol II Degradation by the Viral RdRp

The trimeric IAV viral RNA-dependent RNA polymerase RdRp complex targets cellular RNA Pol II for degradation as a means of altering host gene expression. The RdRP associates with the promoter regions of genes actively transcribed by RNA Pol II, including protein-coding mRNAs and the abundant U snRNAs. Active Pol II transcription and the physical interaction between the CTD of the large subunit of Pol II and the RdRp are required for viral mRNA synthesis by the RdRp [148,149,150]. As mentioned in Section 2.5, the CTD includes a series of tandem repeats that are differentially phosphorylated on Ser2 and Ser5 depending on the stage of transcription [115]. In particular, the RdRp binds to the initiating form of RNA Pol II, bearing phosphate groups on Ser5, which is also the form that interacts with machinery that synthesizes the 5′ 7-methyl guanosine cap of cellular mRNAs [149]. The RdRp-Pol II association has multiple effects. It allows the RdRp to access nascent transcripts. The RdRp then cleaves these RNAs close to the 5′ end to generate short 5′ capped RNA fragments that are used as a primer for viral mRNA transcription, effectively appropriating the 5′ caps of host messages for viral messages, a process known as “cap-snatching” [151]. In addition, it leads to ubiquitination and subsequent degradation of the large subunit of Pol II by the proteasome, with concomitant loss of Pol II transcriptional activity [152,153] (Figure 4). The degradation of Pol II plays a role in circumventing host antiviral response, because it regulates the expression of IFN-stimulated genes like ISG15 [153]. Moreover, Pol II degradation may also contribute to the shift from viral mRNA synthesis to viral genomic RNA synthesis that occurs during the IAV replication cycle. Pol II degradation would naturally reduce the association between RdRp and Pol II, facilitating the shift from viral transcription (which requires Pol II) to viral replication [154]. RdRp-dependent degradation of Pol II has been reported in many strains of IAV, including both human and avian subtypes [152,153,155], suggesting it is a widespread mechanism of transcriptional regulation.

### 3.3. Widespread RNA Degradation by PA-X

A potential role for mRNA degradation in host shutoff by IAV was first reported in 1992 [156]. However, until recently, RNA degradation was believed to be a consequence of the process of cap-snatching by the RdRp subunit PA. In this process, host RNAs are cleaved and the cleaved uncapped RNA fragments are presumably degraded. The recent discovery of PA-X [157], a protein that is produced by a +1 ribosomal frameshift after amino acid 191 of PA [157,158], revealed that cap-snatching and host RNA degradation are two distinct functions. PA and PA-X share the same *N*-terminal RNA endonuclease domain with a PD(D/E)XK nuclease family fold [157,159,160]. PA-X terminates in a short *C*-terminal domain, termed the X-ORF, that is important for the host shutoff activity of PA-X [2,157,161]. Expression of PA-X leads to reduction in reporter and endogenous gene expression [2,157,162], which is attributed to endonucleolytic cleavage and decreased half-life of the RNAs [2] (Figure 2). The X-ORF is either 41 or 61 amino acids (aa), depending on the viral strain. Despite the fact that only the first 15 amino acids of the X-ORF are strictly required for the full extent of PA-X-mediated shutoff of reporter proteins [161], the PA-X variants with longer 61-aa X-ORFs may have stronger activity [163].

PA-X has an important role in reducing the host innate immune responses. In most strains, reducing PA-X production by mutating the frameshift-promoting sequence results in viruses that elicit stronger immune responses in mouse models. This has been observed with pandemic 1918 H1N1 [157], pandemic 2009 H1N1 [164,165], and avian H5N1 [165,166]. Avian H9N2 is an exception, because PA-X loss in this strain reduces host immune responses [167]. In most cases [157,165,166], the stronger immune response triggered by the mutant viruses causes increased pathogenicity of the virus, due to increased lung immunopathology. Of note, the frameshift mutations do not always fully abolish PA-X production [164,166]. The length of the X-ORF (41-aa *vs.* 61-aa) affects pathogenicity of the virus. Altering the length in different viral backgrounds uncovered a correlation between the presence of the longer PA-X, and increased virulence in mice and higher viral titers in human cells [163]. In addition, the length of the tail may be related to host specificity and adaptation, because viruses with the shorter PA-X variants replicate more efficiently in swine cells but less efficiently in human cells [168].

At a molecular level, PA-X is capable of cleaving both ssRNA and dsRNA, whereas PA preferentially cleaves ssRNA [169]. This difference supports the idea that the PA endonuclease activity is dedicated to cleaving pre-mRNAs for the purpose of cap-snatching, and the PA-X endonuclease activity is dedicated to host shutoff. Interestingly, like PA, PA-X also preferentially degrades Pol II-transcribed host RNAs, including ncRNAs, rather than Pol I and Pol III transcripts [2]. Accordingly, PA-X spares viral mRNAs and genomic transcripts that are generated by the viral RdRp, as demonstrated by the fact that the levels of most viral mRNAs and vRNAs are the same in cells infected with wild-type or PA-X-deficient strains of A/PuertoRico/8/1934 H1N1 (PR8) [2]. In fact, mutants that lack the PA-X RNase show lower, not higher, accumulation of viral proteins compared to wild-type infection [2]. This observation suggests that the ability of IAV to degrade host mRNAs allows the virus to access cellular translational machinery preferentially, perhaps because fewer cellular transcripts are available for translation. This may be coupled with other processes that maintain or increase translational function [170]. These data thus support at least a partial role for PA-X in reallocation of cellular machinery. Interestingly, some viral transcripts like M1 are transcribed more robustly in the PR8 PA-X null viruses, while the accumulation of spliced viral transcripts such as M2 and NEP is reduced [2], suggesting that loss of host shutoff by PA-X alters the balance of viral gene expression in multiple ways. At present, we do not yet know how these secondary changes occur.

Surprisingly, PA-X degradation, like the action of NS1, may be coupled to the cellular 3′ end processing in the nucleus. In this respect, PA-X is different from host shutoff RNases from other viruses, like vhs and SOX, that specifically target translating or at least translatable mRNAs in the cytoplasm. Reporter mRNAs that bypass 3′ end processing and end in a hammerhead ribozyme are not degraded by PA-X, even when a templated poly(A) stretch is included to promote translation [2]. Moreover, X-ORF residues that prevent nuclear accumulation of PA-X also prevent its degradation of reporter mRNAs [2]. This mechanism of nuclear target selectivity, unique among the viral RNases, would explain both the preference of PA-X for Pol II transcripts and the escape of viral mRNAs from degradation. Neither the RNAs generated by the other RNA polymerases (RNA Pol I and RNA Pol III) nor viral mRNAs are processed by the cellular Pol II-associated machinery. In contrast, PA-X is similar to several other host shutoff RNases in terms of mechanism of RNA degradation [2], also discussed in Section 2.1. It cleaves RNAs internally, and degradation of fragments is completed by cellular exonucleases, including the 5′-3′ exonuclease Xrn1 [2]. At present, it appears as though the initial cleavage by PA-X can occur anywhere along the length of the mRNA [2].

Another similarity between herpesviral RNases and IAV PA-X are the secondary consequences of PA-X activity (Figure 2). Like herpesviral infection, IAV infection leads to nuclear accumulation of the cytoplasmic poly(A) binding protein PABPC1 [87]. PABPC1 relocalization is PA-X dependent and expression of PR8 PA-X alone also induced a strong nuclear accumulation of PABPC1 [2,87]. As mentioned in Section 2.2., nuclear localization of PABPC1 is accompanied by nuclear retention and hyperadenylation of mRNAs [80,81,87]. Thus, nuclear PABPC could prevent newly transcribed RNAs from replenishing the cytoplasmic pool of mRNAs, further reducing the translatable pool of mRNAs and potentiating host shutoff. Moreover, like in the case of HSV vhs PA-X expression and PABPC1 nuclear localization are accompanied by an inhibition of arsenite-induced stress granule (SG) formation [87]. The mechanism of PA-X-induced SG dissociation and its link to RNA degradation and PABPC relocalization remain to be discovered. Of note, NS1 and the nucleoprotein NP also contribute to SG regulation in IAV infection [87,171], although it is unknown whether this is linked to gene regulation changes.

Interestingly, evidence for conservation of the PA-X protein has been found in almost all IAV strains [172]. Stop codons in the +1 reading frame in the *C*-terminal region of overlap between PA and PA-X (the X-ORF reading frame) are found at two very specific locations in almost all IAV strains [172]. Additionally, the overall rate of synonimous mutations in the 0 reading frame (PA) is low, suggesting evolutionary pressure to maintain the PA-X protein sequence unchanged [172]. Maintaining two functional overlapping reading frames severely constrains the ability of the virus to mutate in this region. Therefore, if PA-X function was not actively retained one would expect to find stop codons at other locations within the X-ORF that do not cause changes to the PA amino acid sequence. This conservation suggests that PA-X-triggered RNA degradation may be a truly ubiquitous host shutoff mechanism for IAV.

### 3.4. Host Shutoff in IAV: One Virus, Multiple Mechanisms, or Different Strains, Different Mechanisms?

In summary (Table 3), at least three different mechanisms of host shutoff are present in IAV-infected cells, but, at present, it is unclear what the relative contributions and the biological roles of these three mechanisms may be. Khaperskyy and McCormick [173] have proposed a temporal division of labor between NS1 and PA-X. Accumulation of NS1 in the nucleus during the initial stages of infection may impair expression of newly transcribed mRNAs, whereas regulation of host mRNAs in the later stages of infection may be mediated by PA-X. Indeed, the low efficiency of ribosomal frameshifting means that sufficient levels of PA-X may not accumulate until later in infection. Pol II degradation may also occur later, to further reduce nascent host RNA production once Pol II activity is no longer needed for RdRp-mediated viral mRNA transcription. Moreover, the RNase activity of PA-X may be able to clear away mRNAs and/or ncRNAs that are already present at the time of infection, whereas the NS1 polyadenylation block and Pol II degradation only affect the fate of newly synthesized RNAs. Another possibility is that different strains of IAV may favor one particular host shutoff mechanism. This is likely true for strains that carry NS1 variants that do not bind CPSF30, and may rely more on PA-X activity and/or Pol II degradation for host shutoff. Given the recent explosion in high-throughput RNA-seq analysis, a comparative analysis of the effects of the three pathways in a transcriptome-wide manner will be extremely useful to understand the relative contribution of the pathways to host gene regulation and importantly to viral replication and pathogenesis.

## 4. Conclusions

Influenza A virus and herpesviruses, specifically those of the α and γ subfamilies, are great examples of the diverse mechanisms viruses employ to carry out host shutoff, and highlight the contribution of host shutoff to enhancing virulence, evading the immune system, abating cellular stress responses, and reallocating machinery. In many ways, it seems counterintuitive that viruses would use such blunt indiscriminate mechanisms to alter cellular physiology, and that they would use multiple versions of them. It is possible that there is greater discrimination of targets during host shutoff than currently appreciated, similarly to what has emerged in the study of KSHV SOX. Another surprising finding is that disparate viruses use similar mechanisms but unrelated viral factors to affect host RNA and protein levels. For example, both IAV and HSV-1 trigger Pol II degradation. RNA-degrading endonucleases are also a common theme among viruses, and we have previously proposed [41] that this may be due to the existence of host pathways that use endonucleolytic cleavage to start RNA degradation, so that virus-induced RNA degradation may not be detected as an aberrant process. Another emerging theme is that host shutoff events may have rippling effects, so that the initiating process, for example RNA degradation, subsequently affects other aspects of gene expression, like nuclear export of mRNAs and transcription in the case of muSOX, creating a feed-forward loop that further potentiates the shutoff. We are just beginning to understand the exact extent of such feedback alterations, but it is interesting that they mirror what happens in the cell under physiological conditions, as recent studies have established that all the steps of gene expression are more interconnected than previously appreciated. Modern tools, such as deep sequencing, have provided new avenues to probe gene expression and have revealed new forms of regulation. These new transcriptome-wide approaches will be very useful in dissecting the temporal and combinatorial effects of the various host shutoff modalities in each virus, and to identify endogenous targets that may further clarify the function of host shutoff in the viral replication cycle and in pathogenesis.

## Figures and Tables

**Table 1:** Host shutoff mechanisms in HSV-1 and -2.

Level of Regulation	Viral Protein	Molecular Activity	Result	Interacting Cellular Proteins
Transcription	Unknown—maybe ICP22 and ICP27	Unknown—maybe dephosphorylation of RNA Pol II?	Inhibition of transcription initiation; inhibition of transcription termination	Unknown—maybe RNA Pol II
RNA processing	ICP27	Inhibits spliceosome formation	Inhibition of splicing; decreased mRNA levels in cytoplasm	SAP145, SRp20, SRPK1
RNA stability	vhs	RNA endonuclease	Degradation of host and viral mRNAs; nuclear relocalization of PABPC	eIF4H, eIF4AI and eIF4AII, TTP

**Table 2:** Host shutoff mechanisms in γ-herpesviruses.

Level of Regulation	Viral Protein	Molecular Activity	Result	Interacting Cellular Proteins
Transcription	MHV68 muSOX (through feedback mechanism)	Unknown	Inhibition of transcription initiation	Xrn1 (functional interaction)
RNA stability	KSHV SOX, MHV68 muSOX, EBV BGLF5	RNA endonuclease	Degradation of host and viral mRNAs; nuclear relocalization of PABPC	Unknown

**Table 3:** Host shutoff mechanisms in influenza A virus.

Level of Regulation	Viral Protein	Molecular Activity	Result	Interacting Cellular Proteins
Transcription	RdRP (PA, PB1, PB2)	Ubiquitination and degradation of large subunit of RNA Pol II	Transcription inhibition	RNA Polymerase II
RNA processing	NS1	Inhibition of mRNA 3′ end cleavage by CPSF and PABPN stimulation of poly(A) addition; interaction with nuclear mRNA export machinery	Inhibition of poly(A) tail addition and nuclear export of mRNA	CPSF30
				PABPN
				Nuclear export proteins (NXF1, p15, Rae1, E1B-AP5, Nup98)
RNA stability	PA-X	RNA endonuclease	Degradation of host mRNA and ncRNAs; nuclear relocalization of PABPC	Unknown
